# 20-year trends in multimorbidity by race/ethnicity among hospitalized patient populations in the United States

**DOI:** 10.1186/s12939-023-01950-2

**Published:** 2023-07-24

**Authors:** Mursal A. Mohamud, David J.T. Campbell, James Wick, Alexander A. Leung, Gabriel E. Fabreau, Marcello Tonelli, Paul E. Ronksley

**Affiliations:** 1 Cumming School of Medicine, Undergraduate Medical Education, University of Calgary, Calgary, AB Canada; 2 Department of Medicine, Cumming School of Medicine, University of Calgary, Calgary, AB Canada; 3 Department of Community Health Sciences, Cumming School of Medicine, University of Calgary, Calgary, AB Canada; 4 Department of Cardiac Sciences, Cumming School of Medicine, University of Calgary, Calgary, AB Canada

**Keywords:** Multimorbidity, Race, Ethnicity, Hospitalization, United States

## Abstract

**Background:**

The challenges presented by multimorbidity continue to rise in the United States. Little is known about how the relative contribution of individual chronic conditions to multimorbidity has changed over time, and how this varies by race/ethnicity. The objective of this study was to describe trends in multimorbidity by race/ethnicity, as well as to determine the differential contribution of individual chronic conditions to multimorbidity in hospitalized populations over a 20-year period within the United States.

**Methods:**

This is a serial cross-sectional study using the Nationwide Inpatient Sample (NIS) from 1993 to 2012. We identified all hospitalized patients aged ≥ 18 years old with available data on race/ethnicity. Multimorbidity was defined as the presence of 3 or more conditions based on the Elixhauser comorbidity index. The relative change in the proportion of hospitalized patients with multimorbidity, overall and by race/ethnicity (Black, White, Hispanic, Asian/Pacific Islander, Native American) were tabulated and presented graphically. Population attributable fractions were estimated from modified Poisson regression models adjusted for sex, age, and insurance type. These fractions were used to describe the relative contribution of individual chronic conditions to multimorbidity over time and across racial/ethnic groups.

**Results:**

There were 123,613,970 hospitalizations captured within the NIS between 1993 and 2012. The prevalence of multimorbidity increased in all race/ethnic groups over the 20-year period, most notably among White, Black, and Native American populations (+ 29.4%, + 29.7%, and + 32.0%, respectively). In both 1993 and 2012, Black hospitalized patients had a higher prevalence of multimorbidity (25.1% and 54.8%, respectively) compared to all other race/ethnic groups. Native American populations exhibited the largest overall increase in multimorbidity (+ 32.0%). Furthermore, the contribution of metabolic diseases to multimorbidity increased, particularly among Hispanic patients who had the highest population attributable fraction values for diabetes without complications (15.0%), diabetes with complications (5.1%), and obesity (5.8%).

**Conclusions:**

From 1993 to 2012, the secular increases in the prevalence of multimorbidity as well as changes in the differential contribution of individual chronic conditions has varied substantially by race/ethnicity. These findings further elucidate the racial/ethnic gaps prevalent in multimorbidity within the United States.

**Prior presentations:**

Preliminary finding of this study were presented at the Society of General Internal Medicine (SGIM) Annual Conference, Washington, DC, April 21, 2017.

**Supplementary Information:**

The online version contains supplementary material available at 10.1186/s12939-023-01950-2.

## Introduction

Multimorbidity is defined as the presence of multiple chronic medical conditions in a single individual. Patients with multimorbidity are more likely to suffer from lower quality of life, poor functionality, and are at risk of premature mortality compared to those without multimorbidity [1–3]. Because of the complex nature of multimorbidity, patients often require the involvement of multiple specialists, polypharmacy, longer hospital stays, and are at a greater risk of readmission [4, 5] – all of which contribute to high health care utilization and costs [6]. The challenges posed by multimorbidity will continue to mount as its prevalence increases, fueled in part by socioeconomic inequities that disproportionately affect certain groups more than others. In the United States (US), these are often racialized populations [7–9]. Furthermore, with increasing life expectancy and an aging population, the burden of multimorbidity is likely to become even greater.

Research indicates there may be differences in the trends and patterns of multimorbidity in certain groups, which may influence their overall health outcomes. These differences are often attributed to sociodemographic, economic, environmental, and behavioral risk factors which can vary substantially across groups [10, 11]. For instance, several studies have described variations in the trends and patterns of multimorbidity based on sex, age, and socioeconomic status [10–16]. Previous studies have also described changes in the prevalence of many individual chronic conditions across racial/ethnic groups [17–19]. Further, the prevalence of multimorbidity has increased with reports indicating that ischemic heart disease, hypertension, diabetes, chronic kidney disease, and heart failure are among the most prevalent chronic conditions among hospitalized adults in the US [20, 21]. However, few studies have fully described the variation in multimorbidity by race/ethnicity. Further, it remains unclear how multimorbidity and the relative contributions of each individual condition have changed differentially by race/ethnicity over time.

In the US, evidence shows that racial and ethnic minorities are at greater risk of morbidity, mortality, and lower quality health care for certain chronic conditions compared to those who are White [22–24]. Such prevalent health inequities make it critical to distinguish and explain variation in the prevalence of multimorbidity and individual chronic conditions across racial/ethnic groups. The objective of our study was to describe trends in multimorbidity by race/ethnicity (i.e., White, Black, Hispanic, Native American, and Asian/Pacific Islander), as well as to determine the patterns and differential contribution of individual chronic conditions to multimorbidity in a hospitalized patient population over a 20-year period within the United States.

## Methods

### Data sources

Data was obtained using the Nationwide Inpatient Sample (NIS) as part of the Healthcare Cost and Utilization Project (HCUP) led by the Agency for Healthcare Research and Quality (AHRQ). This partnership between industry and federal/state authorities was developed to report national and regional estimates of inpatient clinical and resource use [25]. The NIS is a publicly available all-payer inpatient care database containing a stratified sample of discharges (~ 20%) from all US community hospitals each year. Community hospitals are defined by the NIS as “all non-Federal, short-term, general, and other specialty hospitals, excluding hospital units of institutions”. This includes academic medical centers and specialty hospitals such as short-term rehabilitation, obstetric, and pediatric hospitals. Non-community hospitals include institutions such as federal hospitals (e.g., Veterans Administration, Indian Health Service hospitals, and Department of Defense), long-term hospitals, psychiatric hospitals, and hospital units within institutions such as prisons. To account for sampling redesign over time, HCUP provides NIS Trend Weights to allow for the analysis of year-to-year trends. Each de-identified record contains information on patient demographics (e.g., age, sex, race, ethnicity, etc.), primary payer, diagnoses, procedures, length of stay, and hospital characteristics (e.g., hospital ownership, number of beds, teaching status, urban/rural location, and the census division of the hospital) [25]. As this data is publicly available, protected by law and did not contain patient identifiers, the Conjoint Health Research Ethics Board at the University of Calgary deemed this study exempt from ethics review. This study follows the reporting guidelines outlined within the Strengthening the Reporting of Observational Studies in Epidemiology (STROBE) statement [26].

### Exposure

We analyzed annual data on all hospitalized adults (≥ 18 years old) from 1993 to 2012 (20 years). The exposure of interest in our study was race/ethnicity. While ‘race’ and ‘ethnicity’ are often used interchangeably, there are differences between them. Race is a social construct historically used to arbitrarily group populations according to their physical characteristics (e.g., skin color) for social, political, economic, and cultural motivations [27, 28]. However, ethnicity is a much broader construct which is used to encompass cultural and social traditions, ancestry, language, and geographical origins [27, 28]. All participating community hospitals supplied data on the race of hospitalized patients, and some provided both race and ethnicity separately. When both race and ethnicity data were made available, HCUPs databases give precedence to ethnicity (specifically Hispanic) over race in setting the value for a race or ethnicity variable. However, because HCUPs does not distinguish ethnicity and race in each record, both constructs were used to define our exposure. Our final categorization of race/ethnicity included: White, Black, Hispanic, Native American, Asian/Pacific Islander, and other. Hospitalized patients were stratified according to the first five race/ethnic groups (excluding ‘other’ from the analysis).

### Outcome

The primary outcome was multimorbidity defined as the presence of 3 or more conditions as per prior research [29–32]. We used the Elixhauser comorbidity index to define 30 individual chronic conditions within the hospitalization record of each person. These conditions are associated with increased hospital costs, length of stay, and mortality [33, 34] and were determined from hospital discharge records using the International Classification of Diseases, Ninth Revision, Clinical Modification (ICD-9-CM).

### Other variables of interest

In this study, demographic variables such as age and sex were included. Age was categorized as 18–44, 45–64, 65–74, and 75 or over which are well-defined cut-points in health services research and roughly reflect quartiles of the age distribution. Sex was categorized by the NIS as either male or female [35], and throughout this study, the term ‘sex’ is used to refer to biological and physiological differences between males and females, such as differences in reproductive organs and sex chromosomes.

The NIS also provides insurance type, or the expected primary payer (Medicare, Medicaid, Private Insurance, and Other). Medicaid is a public health insurance for some low-income families, pregnant women and children, the elderly, and people with disabilities [36]. Medicare is also a public health insurance program for people age 65 or older, some younger people with disabilities and people with end stage renal disease and amyotrophic lateral sclerosis (ALS) [37]. Because the primary payer was missing for a small proportion of hospitalized patients (0.3%), the insurance type variable was constructed using other general groups as outlined in the NIS data dictionary [38]. For instance, “Medicaid” includes both fee-for-service and Medicaid coverage plans managed by private companies. “Medicare” also includes both fee-for-service and Medicare coverage plans managed by private companies. “Private insurance” included commercial carriers, private health maintenance organizations and preferred provider organizations. The category of “Other” included all other government programs such as Worker’s compensation, Title V, and CHAMPUS.

### Statistical analysis

Descriptive statistics were used to determine the relative change in the proportion of hospitalized patients with multimorbidity both overall and stratified by race/ethnicity. Hospitalization data were analyzed separately by year (1993–2012) then blocked into 5-year bands and reported as percent change. Changes in the overall number of chronic conditions among hospitalized patients was displayed graphically by year. Additionally, line graphs were used to show the proportion of hospitalized patients with multimorbidity across all race/ethnicity groups stratified by age, in both 1993 and 2012.

We used population attributable fractions (PAF) to describe the relative influence of individual chronic conditions within the construct of multimorbidity over time and across racial/ethnic groups [39, 40]. PAF represents the proportion of multimorbidity that (theoretically) would not occur if the individual chronic condition was not present in the population. To allow for estimation of PAF, we first created multivariable modified Poisson models. Based on descriptive statistics and univariate Poisson modelling, we then created final models for each 5-year block including multimorbidity as the dependent variable and all comorbidities, with age, sex, and insurance type as covariates. The PAF for individual conditions was displayed graphically using circle plots, where the relative size of the circles indicate the relative contribution of each condition within each year for each race/ethnicity (truncated to top 15 conditions). All statistical analyses were conducted in STATA 15 (College Station, TX: StataCorp LLC).

## Results

Our data included 123,613,970 unique hospitalizations between 1993 and 2012. Over time, the average age of hospitalized patients increased (Table 1). The earliest 5-year block (1993–1997) found that the average proportion of hospitalized patients aged 45–64 was 22.3%, but this increased to 29.2% in the latest 5-year block (2008–2012). There was generally no major change in the sex of hospitalized patients during the observation period. The proportion of patients who received Medicare or Medicaid increased slightly during the 20-year period, with an increase of 3.9% and 2.3% respectively. In contrast, the proportion of privately insured hospitalized patients decreased by 6.6%. The racial/ethnic distribution also changed over the 20-year period with a decrease in the overall proportion of White hospitalized patients and increases in all other racial/ethnic groups, most notably in Hispanic populations (Figures S1-4). The prevalence of uncomplicated hypertension increased the most of all chronic conditions (+ 21.1%) over the 20-years, followed by depression (+ 11.0%), renal failure (+ 10.4%), and chronic pulmonary disease (+ 9.9%). Conversely, AIDS/HIV, metastatic cancer, and solid tumors without metastasis all decreased over the 20-year period.

**Table 1:** Changes in demographic and clinical characteristics over time

	1993–1997		1998–2002		2003–2007		2008–2012		1993 to 2012
	(n = 27,169,722)		(n = 30,339,475)		(n = 33,155,380)		(n = 32,949,393)		% change
	***n***	***%***	***n***	***%***	***n***	***%***	***n***	***%***	
**Age**									
Mean (SD)	55.9 (21.4)		56.8 (21.2)		56.7 (21.1)		57.3 (20.8)		
Median (IQR)	59 (36–74)		59 (38–75)		58 (38–75)		59 (40–75)		
18–44	9,500,794	35.0	9,889,714	32.6	10,508,890	31.7	9,719,897	29.5	-7.1
45–64	6,067,284	22.3	7,429,314	24.5	8,993,160	27.2	9,608,625	29.2	7.2
65–74	4,915,581	18.1	5,028,732	16.6	5,093,150	15.4	5,285,204	16.1	-1.8
75+	6,678,417	24.6	7,985,391	26.3	8,511,332	25.7	8,295,500	25.2	1.6
**Sex**									
Male	10,685,865	39.3	11,742,697	38.7	12,873,798	38.9	13,236,534	40.2	1.0
Female	16,480,719	60.7	18,592,333	61.3	20,181,689	61.1	19,654,495	59.8	-1.0
**Race**	(n = 21,544,280)		(n = 22,735,155)		(n = 24,377,850)		(n = 28,885,723)		
White	16,479,011	76.5	16,620,980	73.1	16,903,419	69.3	19,906,573	68.9	-6.0
Black	2,813,648	13.1	2,997,642	13.2	3,335,655	13.7	4,191,906	14.5	4.1
Hispanic	1,569,632	7.3	2,050,650	9.0	2,785,390	11.4	3,001,576	10.4	4.5
Asian or Pacific Islander	289,395	1.3	451,594	2.0	550,302	2.3	682,225	2.4	1.4
Native American	56,313	0.3	70,572	0.3	124,302	0.5	194,451	0.7	0.5
Other	336,281	1.6	543,717	2.4	678,782	2.8	908,992	3.1	1.5
**Primary Payer**									
Medicare	11,796,540	43.4	13,370,051	44.1	14,719,684	44.4	14,876,249	45.1	3.9
Medicaid	3,557,888	13.1	3,813,173	12.6	4,697,822	14.2	4,986,132	15.1	2.3
Private including HMO	9,306,011	34.3	10,652,293	35.1	10,760,790	32.5	9,934,117	30.1	-6.6
Other	2,379,766	8.8	2,395,003	7.9	2,916,863	8.8	3,075,694	9.3	0.2
**Elixhauser Comorbidities**									
AIDS/HIV	230,715	0.8	168,850	0.6	170,334	0.5	149,653	0.5	-0.5
Alcohol abuse	1,246,608	4.6	1,352,337	4.5	1,567,882	4.7	1,810,355	5.5	1.5
Blood loss anemia	406,186	1.5	443,222	1.5	517,789	1.6	397,418	1.2	-0.2
Cardiac arrythmias	3,670,598	13.5	4,454,579	14.7	5,371,365	16.2	6,148,003	18.7	8.2
Chronic pulmonary disease	3,890,058	14.3	5,040,962	16.6	6,530,526	19.7	7,067,866	21.5	9.9
Coagulopathy	445,965	1.6	623,161	2.1	856,071	2.6	1,269,182	3.9	3.4
Heart failure	3,332,632	12.3	4,083,039	13.5	4,673,433	14.1	4,644,853	14.1	3.5
Deficiency anemia	380,993	1.4	476,310	1.6	632,612	1.9	890,402	2.7	1.8
Depression	1,381,748	5.1	2,132,557	7.0	3,101,654	9.4	4,196,257	12.7	10.4
Diabetes with complications	944,087	3.5	1,102,217	3.6	1,317,941	4.0	1,544,587	4.7	1.0
Diabetes without complications	2,908,635	10.7	4,151,389	13.7	5,245,770	15.8	6,046,590	18.4	11.0
Drug abuse	814,703	3.0	922,268	3.0	1,296,003	3.9	1,543,072	4.7	2.6
Fluid and electrolyte disorders	3,947,372	14.5	4,298,265	14.2	5,759,900	17.4	7,198,154	21.8	10.3
Hypertension (Complications)	1,012,064	3.7	1,407,058	4.6	2,180,686	6.6	3,610,658	11.0	9.5
Hypertension (Uncomplicated)	5,854,638	21.5	8,773,827	28.9	11,365,544	34.3	12,373,099	37.6	21.1
Hypothyroidism	1,091,362	4.0	1,823,771	6.0	2,516,676	7.6	3,362,677	10.2	8.5
Liver disease	511,243	1.9	684,954	2.3	971,289	2.9	1,291,010	3.9	2.8
Lymphoma	238,356	0.9	264,031	0.9	305,388	0.9	342,741	1.0	0.2
Metastatic cancer	1,062,218	3.9	929,280	3.1	968,550	2.9	984,424	3.0	-1.1
Obesity	683,048	2.5	1,132,672	3.7	1,917,909	5.8	3,130,289	9.5	9.7
Other neurological disorders	1,387,585	5.1	1,653,846	5.5	1,941,674	5.9	2,419,531	7.3	3.8
Paralysis	648,845	2.4	414,692	1.4	412,789	1.2	533,673	1.6	-0.4
Peptic ulcer disease excluding bleeding	312,792	1.2	294,334	1.0	272,056	0.8	278,181	0.8	-0.1
Peripheral vascular disorders	1,098,796	4.0	1,350,332	4.5	1,595,583	4.8	1,881,090	5.7	2.9
Psychoses	617,981	2.3	688,462	2.3	879,623	2.7	950,439	2.9	0.7
Pulmonary circulation disorders	350,189	1.3	504,127	1.7	704,896	2.1	1,151,164	3.5	3.0
Renal failure	940,027	3.5	1,304,618	4.3	2,324,209	7.0	3,835,816	11.6	10.4
Rheumatoid arthritis/collagen vascular diseases	479,137	1.8	633,590	2.1	779,436	2.4	879,591	2.7	1.5
Solid tumor without metastasis	1,739,872	6.4	1,748,258	5.8	2,180,072	6.6	1,992,505	6.0	-0.7
Valvular disease	1,428,958	5.3	1,624,206	5.4	1,883,620	5.7	1,780,169	5.4	1.5
**Number of Comorbidities**									
0	8,542,050	31.4	8,471,718	27.9	7,800,679	23.5	6,292,038	19.1	-16.4
1	6,152,596	22.6	6,297,214	20.8	6,034,422	18.2	5,179,642	15.7	-9.3
2	5,494,607	20.2	6,130,892	20.2	6,491,272	19.6	5,892,560	17.9	-3.7
≥ 3	6,980,469	25.7	9,439,651	31.1	12,829,007	38.7	15,585,153	47.3	29.5

Abbreviations: AIDS/HIV: Acquired Immunodeficiency Syndrome/Human immunodeficiency virus; HMO: Health Maintenance Organizations; IQR: Interquartile Range; SD: Standard Deviation

### Prevalence of multimorbidity and trends in individual chronic conditions by race/ethnicity

The prevalence of multimorbidity increased in all racial/ethnic groups, particularly in White, Black, and Native American populations which saw relative increases of + 29.4%, + 29.7%, and + 32.0% between 1993 and 2012 respectively (Table 2). Hispanic and Asian or Pacific Islander populations also exhibited an increase in the prevalence of multimorbidity over this period (23.0% and 23.5%, respectively). As of 2012, the overall prevalence of multimorbidity was 54.8% for Blacks, 52.8% for Whites, 50.4% for Native Americans, 39.2% for Hispanics, and 38.0% for Asian or Pacific Islanders.

**Table 2:** Demographic and clinical characteristics, by race/ethnicity

	White			Black			Hispanic			Asian or Pacific Islander			Native American		
	1993	2012	1993 to 2012	1993	2012	1993 to 2012	1993	2012	1993 to 2012	1993	2012	1993 to 2012	1993	2012	1993 to 2012
	(n = 3,126,425)	(n = 4,001,210)	% change	(n = 520,442)	(n = 853,795)	% change	(n = 273,784)	(n = 597,701)	% change	(n = 47,094)	(n = 138,399)	% change	(n = 10,103)	(n = 40,526)	% change
**Age**															
18–44	31.2	23.9	-7.3	53.4	36.1	-17.3	55.5	47.6	-7.9	54.4	42.8	-11.6	53.9	38.4	-15.5
45–64	22.0	28.8	6.8	23.5	35.9	12.4	20.2	26.1	5.9	19.4	21.9	2.5	23.3	31.6	8.3
65–74	20.2	18.4	-1.8	11.9	13.4	1.5	11.7	11.5	-0.2	12.8	13.6	0.8	12.0	14.4	2.4
75+	26.5	29.0	2.5	11.2	14.6	3.4	12.6	14.9	2.3	13.4	21.8	8.4	10.8	15.6	4.8
**Sex**															
Male	41.0	42.1	1.1	36.9	39.2	2.3	34.8	35.8	1.0	31.5	33.0	1.5	43.1	38.3	-4.8
Female	59.0	57.9	-1.1	63.1	60.8	-2.3	65.2	64.2	-1.0	68.5	67.0	-1.5	56.9	61.7	4.8
**Primary Payer**															
Medicare	47.7	51.8	4.1	28.7	39.7	11.0	23.7	29.5	5.8	19.6	32.3	12.7	25.0	36.7	11.7
Medicaid	7.9	10.4	2.5	30.4	27.0	-3.4	33.6	32.7	-0.9	26.1	19.7	-6.4	26.0	23.6	-2.4
Private including HMO	36.2	29.5	-6.7	26.5	21.7	-4.8	27.3	22.6	-4.7	44.1	39.4	-4.7	30.1	23.1	-7.0
Other	8.2	8.2	0.0	14.4	11.6	-2.8	15.4	15.2	-0.2	10.1	8.7	-1.4	18.9	16.7	-2.2
**Comorbidities**															
AIDS/HIV	0.5	0.2	-0.3	3.2	1.5	-1.7	2.3	0.6	-1.7	0.3	0.1	-0.2	1.1	0.2	-0.9
Alcohol abuse	4.0	5.8	1.8	8.3	6.8	-1.5	4.8	6.0	1.2	1.3	2.1	0.8	11.1	12.2	1.1
Blood loss anemia	1.4	1.1	-0.3	1.7	1.4	-0.3	1.1	1.1	0.0	1.5	1.2	-0.3	1.0	1.3	0.3
Cardiac arrythmias	14.3	23.1	8.8	7.3	16.0	8.7	7.3	12.2	4.9	7.5	15.7	8.2	6.7	14.9	8.2
Chronic pulmonary disease	14.3	24.8	10.5	9.2	22.6	13.4	8.4	14.2	5.8	6.4	13.1	6.7	9.8	20.1	10.3
Coagulopathy	1.5	4.9	3.4	1.5	4.4	2.9	1.5	4.6	3.1	1.5	5.1	3.6	1.7	5.8	4.1
Heart failure	12.1	15.2	3.1	9.7	16.9	7.2	7.0	9.6	2.6	6.4	10.6	4.2	7.8	12.6	4.8
Deficiency anemia	1.2	2.9	1.7	2.2	4.1	1.9	1.2	2.7	1.5	1.2	2.7	1.5	1.3	3.5	2.2
Depression	4.6	16.2	11.6	3.2	10.5	7.3	3.3	9.7	6.4	1.8	5.7	3.9	4.2	13.4	9.2
Diabetes with complications	3.9	4.6	0.7	6.8	7.3	0.5	5.2	6.4	1.2	3.3	5.5	2.2	7.2	8.0	0.8
Diabetes without complications	8.3	18.8	10.5	9.6	23.1	13.5	7.5	19.9	12.4	6.9	18.4	11.5	8.9	22.9	14.0
Drug abuse	1.8	4.7	2.9	7.9	8.8	0.9	4.0	4.9	0.9	0.7	1.8	1.1	6.4	6.7	0.3
Fluid and electrolyte disorders	14.9	25.0	10.1	15.9	25.8	9.9	11.0	19.6	8.6	12.7	21.5	8.8	11.5	25.4	13.9
Hypertension (Complications)	2.9	11.6	8.7	6.4	19.2	12.8	3.0	11.3	8.3	2.9	13.2	10.3	2.8	12.5	9.7
Hypertension (Uncomplicated)	19.0	41.1	22.1	20.1	38.5	18.4	12.7	28.8	16.1	13.1	29.4	16.3	12.1	34.1	22.0
Hypothyroidism	3.7	13.7	10.0	1.3	5.6	4.3	1.3	7.5	6.2	1.2	7.1	5.9	1.8	10.0	8.2
Liver disease	1.7	4.3	2.6	1.9	4.2	2.3	2.3	5.9	3.6	1.7	4.4	2.7	3.6	7.7	4.1
Lymphoma	1.0	1.2	0.2	0.6	1.0	0.4	0.6	0.8	0.2	0.7	1.0	0.3	0.6	0.8	0.2
Metastatic cancer	4.8	3.3	-1.5	3.1	3.0	-0.1	2.5	2.3	-0.2	4.0	3.7	-0.3	2.0	2.3	0.3
Obesity	2.1	11.6	9.5	2.7	14.4	11.7	1.6	11.3	9.7	0.6	4.9	4.3	2.2	13.4	11.2
Other neurological disorders	4.7	8.6	3.9	5.4	9.4	4.0	3.4	6.1	2.7	2.7	5.8	3.1	4.2	7.6	3.4
Paralysis	2.2	1.7	-0.5	2.6	2.2	-0.4	1.7	1.5	-0.2	2.6	1.7	-0.9	1.7	1.7	0.0
Peptic ulcer disease excluding bleeding	1.1	0.9	-0.2	1.0	0.9	-0.1	0.8	0.8	0.0	1.1	1.1	0.0	0.9	0.8	-0.1
Peripheral vascular disorders	4.1	6.9	2.8	2.9	5.8	2.9	1.8	4.4	2.6	1.5	4.4	2.9	2.3	5.3	3.0
Psychoses	2.3	2.7	0.4	3.7	5.5	1.8	2.6	2.8	0.2	1.5	2.3	0.8	1.8	2.5	0.7
Pulmonary circulation disorders	1.2	4.3	3.1	0.9	4.8	3.9	0.6	2.4	1.8	0.5	2.7	2.2	0.7	3.2	2.5
Renal failure	2.6	12.6	10.0	6.3	19.4	13.1	3.0	11.8	8.8	3.7	13.9	10.2	4.2	13.6	9.4
Rheumatoid arthritis/collagen vascular diseases	1.6	3.1	1.5	1.5	3.0	1.5	1.0	2.2	1.2	1.1	1.9	0.8	1.4	3.4	2.0
Solid tumor without metastasis	7.5	6.4	-1.1	5.0	5.4	0.4	4.1	4.5	0.4	6.2	7.1	0.9	3.6	4.5	0.9
Valvular disease	5.3	7.0	1.7	2.7	4.3	1.6	2.6	3.3	0.7	2.9	4.4	1.5	1.8	4.3	2.5
**Number of Comorbidities**															
0	32.0	15.3	-16.7	33.1	15.7	-17.4	47.7	30.9	-16.8	52.6	33.2	-19.4	38.5	19.4	-19.1
1	24.0	14.6	-9.4	21.3	13.5	-7.8	20.5	15.3	-5.2	19.2	14.8	-4.4	25.0	14.3	-10.7
2	20.5	17.3	-3.2	20.5	16.0	-4.5	15.5	14.6	-0.9	13.7	14.1	0.4	18.1	15.9	-2.2
≥ 3	23.4	52.8	29.4	25.1	54.8	29.7	16.2	39.2	23.0	14.5	38.0	23.5	18.4	50.4	32.0

Abbreviations: AIDS/HIV: Acquired Immunodeficiency Syndrome/Human immunodeficiency virus; HMO: Health Maintenance Organizations

With respect to individual chronic conditions, uncomplicated hypertension had the largest relative increase in proportion across all groups, particularly among White (+ 22.1%) and Native American (+ 22.0%) hospitalized patients. The relative proportion of hospitalized patients with chronic pulmonary disease, renal failure, complicated hypertension, and heart failure (HF) also increased in all groups. However, the greatest relative increases in these conditions were observed among Black patients (+ 13.4, + 13.1, + 12.8 and + 7.2% respectively). By 2012, Black patients had the highest overall prevalence of renal failure (19.4%), complicated hypertension (19.2%), and heart failure (16.9%). In contrast, White populations had the highest prevalence of hypothyroidism (13.7%) and depression (16.2%) in 2012.

### Trends in multimorbidity

**Fig. 1:**
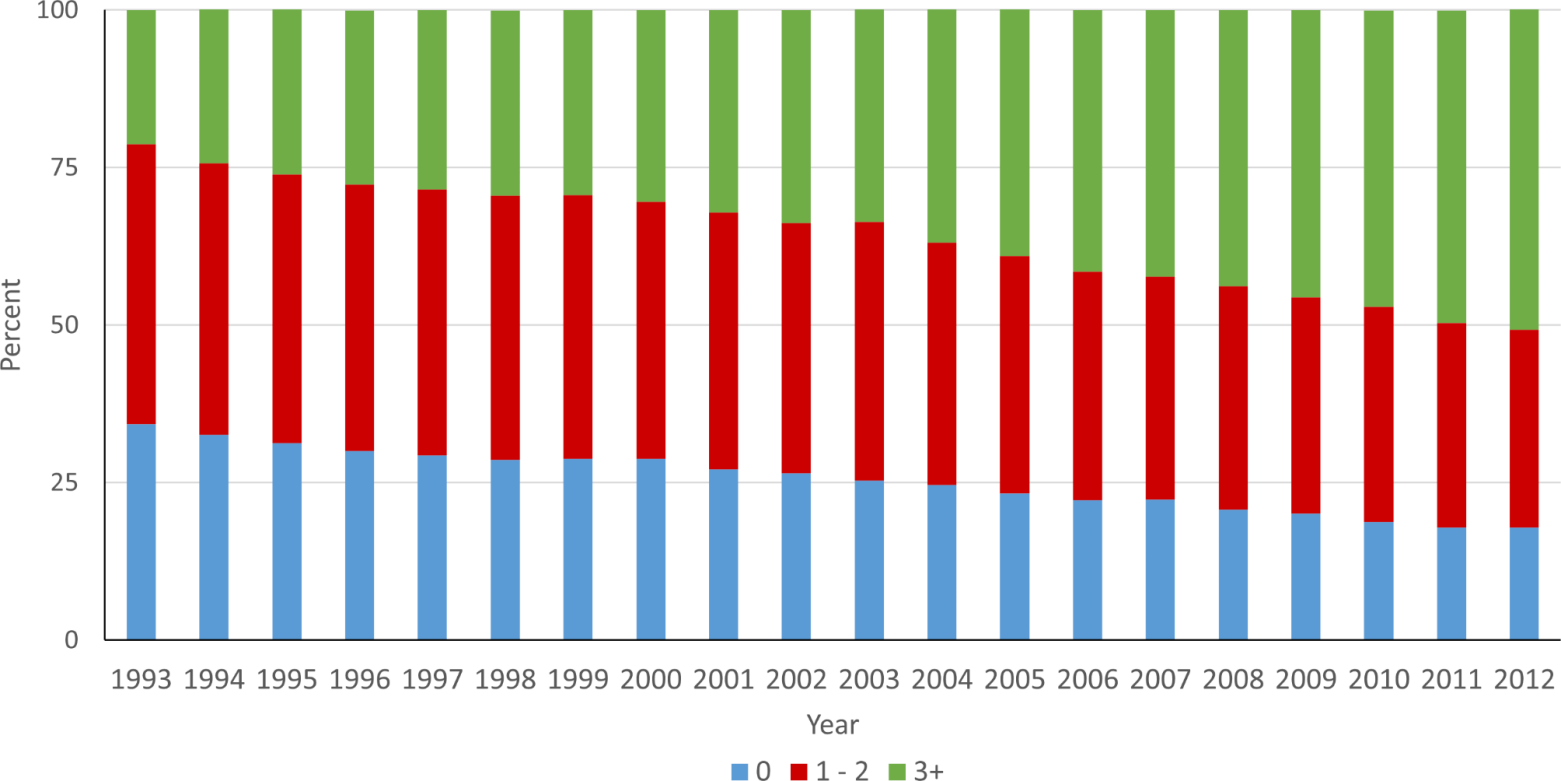
Number of Elixhauser comorbidities, by year

**Fig. 2:**
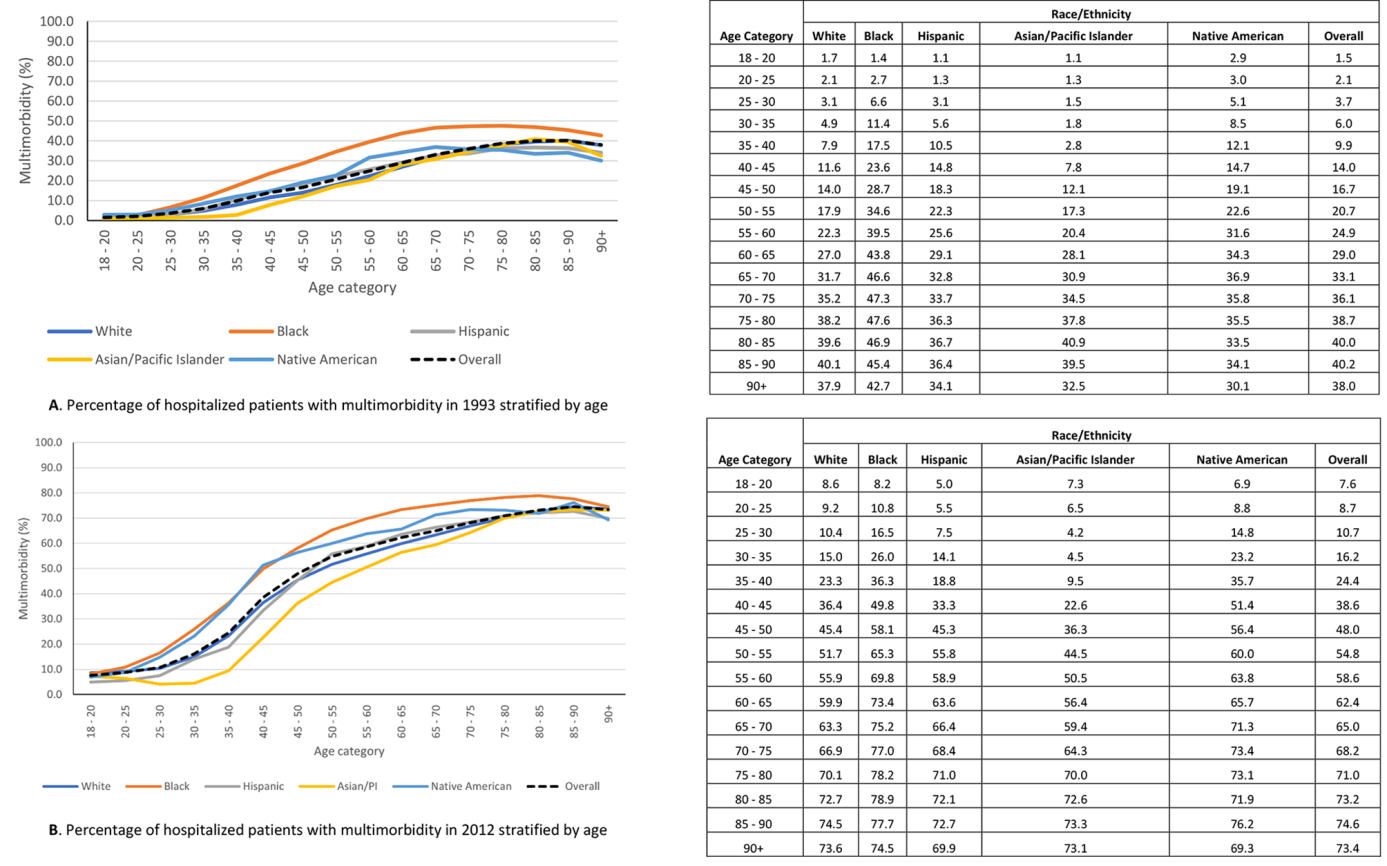
**A.** Percentage of hospitalized patients with multimorbidity in 1993 stratified by age. **B.** Percentage of hospitalized patients with multimorbidity in 2012 stratified by age

The relative proportion of hospitalized patients with 2 or fewer chronic conditions has decreased while the proportion of those with 3 or more has increased (Fig. 1). Further the overall prevalence of multimorbidity increased over the 20-year timeframe for all racial/ethnic groups (**Figure S5**). Multimorbidity is known to increase with advancing age, and this was demonstrated in our data, however we noted that at all ages, both in 1993 and in 2012, Black hospitalized patients had the highest prevalence of multimorbidity (Fig. 2A and B).

### Population attributable fraction

**Fig. 3:**
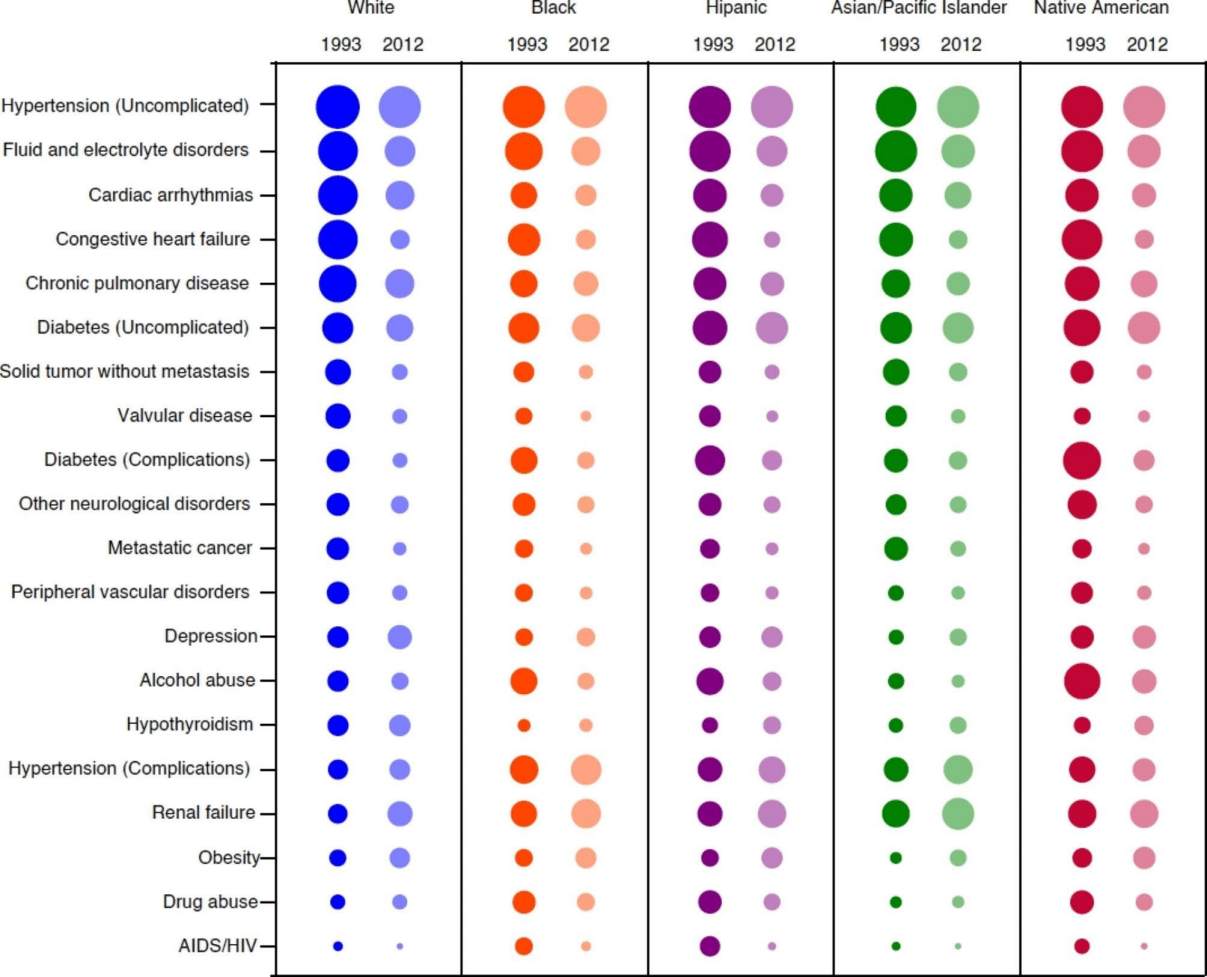
Population attributable fraction for multimorbidity (i.e., the relative influence of individual morbidities on the outcome of multimorbidity) by year and race/ethnicity

Over the 20-year period the relative contribution of uncomplicated hypertension to multimorbidity increased in all race/ethnic groups, particularly among Hispanic hospitalized patients (+ 5.8%), becoming the leading contributor to multimorbidity for all race/ethnic groups (Fig. 3, Table S1 and Table S2). Hispanic hospitalized patients also had the highest PAF values for diabetes without complications (15.0%), diabetes with complication (5.1%), and obesity (5.8%). In fact, over the 20-year period, Hispanic hospitalized patients were the only group that experienced an increase in the contribution of diabetes without complication (+ 0.9%).

The contribution of depression to multimorbidity was highest for White hospitalized patients (7.0%), and lowest among Asian/Pacific Islanders (3.4%). The contribution of drug abuse to multimorbidity affected Black hospitalized patients (3.8%) and Hispanic hospitalized patients (3.3%) the most despite its contribution decreasing over time in these groups (-2.6% and − 2.4% respectively). Conversely, the contribution of drug abuse to multimorbidity increased among White hospitalized patients by 0.3%. We also saw large increases in the PAF for renal failure across all groups, though the largest relative changes were observed in Hispanic (+ 4.4%) and Asian/Pacific Islander (+ 4.5%) populations. The PAF for HF and cardiac arrhythmias decreased substantially for all groups, but most among White (-14.5% and − 8.4%, respectively) and least among Black populations (-8.8% and − 3.0%, respectively). (Table S1).

## Discussion

Our study explored trends and patterns in multimorbidity by race/ethnicity over a 20-year period (1993–2012) among hospitalized patients in the US. We were able to describe trends in the prevalence of multimorbidity as well as individual disease contributions to multimorbidity, stratified by race/ethnicity. Our study adds to the multimorbidity literature by highlighting racial/ethnic variations in multimorbidity trends, and more uniquely, highlighting changes in specific chronic disease contributions among hospitalized populations. Overall, we determined that (1) from 1993 to 2012, secular increases in the prevalence of multimorbidity have differed by race/ethnicity; (2) Black hospitalized patients continued to have a higher prevalence of multimorbidity compared to all other race/ethnic groups; (3) the contribution of cardiometabolic diseases to multimorbidity has increased, particularly among Hispanic patients; and (4) regardless of race/ethnicity, the prevalence of HF has increased while its contribution to multimorbidity has decreased. Our findings provide insight into the differential patterns of chronic conditions that contribute to multimorbidity across racial/ethnic communities in the United States. This is particularly important as many of the leading contributors to multimorbidity (e.g., obesity, hypertension, diabetes, and their related complications) are modifiable and potentially preventable through individual education, modifications to environments and opportunities to promote overall health [41–43].

The major increase in the prevalence of multimorbidity over time (irrespective of race/ethnic) amongst hospitalized patients is consistent with previous studies that have demonstrated similar trends in the general US population [44]. However, we also examined the relative contribution of individual conditions to multimorbidity which can help inform disease-targeted preventative strategies for specific conditions in the general population and in at-risk racial/ethnic groups. For example, across all groups we observed an increase in the prevalence of uncomplicated hypertension, fluid and electrolyte disorders, chronic pulmonary disease, diabetes without complication, obesity, and renal failure. From our PAF-analysis, we also determined that these chronic conditions generally had some of the highest contributions to multimorbidity. The fact that these conditions are highly prevalent and increasing over time, likely contributed to the increase in multimorbidity during the 20-year period. The racial/ethnic variations in the prevalence of individual conditions also explains the differences in multimorbidity estimates by race/ethnicity. Focused initiatives should be implemented to reduce exposure to the modifiable risk-factors associated with these highly prevalent conditions. It is also crucial to include members of the affected communities in the development, implementation, and evaluation of such initiatives as community participation has been shown to lead to programs and policies that are more likely to improve outcomes [45].

Consistent with previous studies within the general population, we found that Black hospitalized patients had a higher prevalence of multimorbidity compared to all other race/ethnic groups [12, 46]. Previous studies have attributed these disparities to the socioeconomic and structural inequities faced by the Black population. We found evidence of this as Black and Hispanic hospitalized patients had the highest prevalence of Medicaid – which has been used in previous studies as a proxy for individual SES [47–49]. Furthermore, many Black neighborhoods in the U.S predispose individuals to greater levels of poverty, environmental and psychosocial stress, higher rates of food insecurity, limited access to a healthy diet, and lower quality health care [50, 51]. These environmental conditions are the product of a history of systemic exclusion and discrimination of Black Americans that have prevented them from achieving social, material, and economic control [52]. Therefore, these upstream inequities have predisposed Black Americans to otherwise preventable conditions (e.g., obesity, diabetes, hypertension, etc.) much earlier in their life-course. Such predispositions and sustained inequities have also been associated with worse health outcomes later in life [53, 54]. To address the disparities of multimorbidity among Black hospitalized patients, it is imperative to also address the upstream socioeconomic inequities associated with adverse health outcomes. If these social determinants are not addressed, Black populations in the U.S. will continue to face more adverse health outcomes.

We also found that the contribution of cardiometabolic diseases has increased significantly across all race/ethnic groups, particularly among Hispanic hospitalized patients. Despite having the second lowest prevalence of multimorbidity, Hispanic patients had the greatest contribution from diabetes, hypertension, and obesity. Distinctly, the contribution of diabetes without complication has decreased in all race/ethnic groups with the exception of Hispanic hospitalized patients. Previous studies have also described higher rates of metabolic conditions among Hispanics [55, 56]. Our findings suggest that multimorbidity among Hispanic patients may be attributed to a higher susceptibility to these particular conditions. Therefore, Hispanic populations may significantly benefit from preventive programs aimed at cardiometabolic risk factors to ultimately reduce the risk of multimorbidity [42, 43, 57].

Consistent with previous studies, we saw an overall increase in the prevalence of HF amongst hospitalized patients [58, 59]. This could be attributed to the rise of other non-HF comorbidities, resulting in more medically complex HF patients [60]. This is supported by our PAF analysis which indicates that while the contribution of HF has decreased, the contributions of conditions such as obesity, hypertension, and renal failure has substantially increased. This decrease in the relative HF-contribution to multimorbidity is most likely due to improvements in clinical management of coronary artery disease (CAD) and HF that have reduced the overall incidence/prevalence of severe HF [61]. However, it is also important to note that the decrease in HF contribution was not equal across all racial/ethnic groups. Our findings show that while White hospitalized patients experienced the greatest reduction in HF contribution to multimorbidity, Black hospitalized patients experienced the least. Additionally, Black hospitalized patients went from having the lowest HF-contribution in 1993, to having the highest in 2012. This finding supports previous studies that show Black populations experience higher prevalence and worse outcomes of HF compared to White population [62, 63]. Such racial/ethnic differences may be due to variations in clinical decision-making, insurance coverage, and access to appropriate treatments [64, 65].

There are several strengths of our study. First, this study quantitatively describes, over a 20-year period, the contribution of individual chronic conditions to the burden of multimorbidity in hospitalized patients by race/ethnicity, which is a valuable addition to the existing literature. Second, past multimorbidity studies on race/ethnicity typically only consider White, Black, and sometimes Hispanic populations, while often ignoring or combining Asian/Pacific Islander and Native American populations into another racial/ethnic category often due of issues of small sample sizes within these populations [22, 46, 66–68]. However, the NIS is an administrative database designed to be representative of all non-federal community hospitals in the US and provides large sample sizes that allow for in-depth and granular analyses across diverse racial/ethnic groups.

However, our study should be interpreted in light of its limitations. As with any administrative database that relies on coded hospital information, the NIS is susceptible to misclassification and/or changes in coding practices over time. Therefore, the increase in multimorbidity could be a function of systematic up-coding within hospital data. However, the NIS provides trend weights to account for temporal variations in coding practice to allow for year-to-year comparisons. While our analysis does not include more contemporary data, there were important changes to the NIS sampling strategy after 2012 that would result in spurious changes to prevalence estimates within studies reporting trends over time [69]. It is also important to note that our study was limited to hospitalized patient samples, therefore our findings can only be generalized to hospital settings due to the differences in the types of comorbidities prevalent between hospitalized patients and outpatients. Furthermore, with administrative data, the more health care encounters a patient has, the more likely they are to accrue diagnoses that will apply to future encounters. Therefore, our analyses may underestimate the true level of multimorbidity for marginalized groups (e.g., racial/ethnic minorities) with less access to care. While the Affordable Care Act (i.e., Obamacare) did expand coverage to those who were previously uninsured (which have over-representation from racial/ethnic minorities), this program was enacted in 2010 and wide coverage was not made available until a few years after [70]. Finally, we recognize that race may be a poor surrogate for other complex social constructs [71, 72] and may not fully capture the heterogeneity in the social, political, economic, and environmental circumstances between and within the defined racial/ethnic groups. However, the deep historical context of race and its impact on contemporary institutions within the United States, make it a critical variable to measure in order to understand the health inequities present within health care and medical research.

In summary, our study highlights the growing burden of multimorbidity among hospitalized populations in the United States and how this has changed differentially across 5 different racial/ethnic groups over a 20-year period. We were also able to identify individual chronic conditions that contribute differently to the construct of multimorbidity across racial/ethnic groups. These findings further elucidate the racial/ethnic gaps prevalent in multimorbidity within the United States. It also provides insight into the unique health inequalities faced by different racial/ethnic communities.

## Electronic supplementary material

Below is the link to the electronic supplementary material.

Supplementary Material 1

## Data Availability

We are not able to make our data set available to other researchers due to our contractual arrangements with the data custodian (US Agency for Healthcare Research and Quality). Requests for similar data sources can be made at: https://www.hcup-us.ahrq.gov/tech_assist/centdist.jsp.
