# Supplementary material for: 20-year trends in multimorbidity by race/ethnicity among hospitalized patient populations in the United States
Source: Int J Equity Health. 2023 Jul 24;22:137. doi: 10.1186/s12939-023-01950-2 (PMC10367428; doi:10.1186/s12939-023-01950-2)
Supplement: Supplementary file 1 — Supplementary Material 1 [file 12939_2023_1950_MOESM1_ESM.docx]

Table S1. Population attributable fractions (expressed as percent) for 30 Elixhauser comorbidities, 1993 and 2012, by race/ethnicity

| **Comorbidity** | **White** | | | **Black** | | | **Hispanic** | | | **Asian/ Pacific Islander** | | | **Native American** | | |
| --- | --- | --- | --- | --- | --- | --- | --- | --- | --- | --- | --- | --- | --- | --- | --- |
|  | 1993 | 2012 | Diff | 1993 | 2012 | Diff | 1993 | 2012 | Diff | 1993 | 2012 | Diff | 1993 | 2012 | Diff |
| Hypertension (Uncomplicated) | 21.16 | 24.06 | 2.90 | 24.14 | 26.17 | 2.03 | 21.55 | 27.31 | 5.75 | 23.75 | 26.75 | 3.00 | 18.51 | 22.62 | 4.11 |
| Fluid and electrolyte disorders | 18.83 | 11.86 | -6.97 | 19.00 | 11.38 | -7.62 | 20.71 | 13.74 | -6.97 | 25.58 | 16.18 | -9.40 | 18.53 | 13.39 | -5.14 |
| Cardiac arrhythmias | 18.73 | 10.38 | -8.35 | 8.60 | 5.61 | -2.99 | 13.23 | 6.93 | -6.31 | 15.35 | 9.81 | -5.54 | 11.21 | 6.45 | -4.76 |
| Heart failure | 18.62 | 4.10 | -14.52 | 13.54 | 4.75 | -8.79 | 15.29 | 2.95 | -12.34 | 15.94 | 4.10 | -11.84 | 17.16 | 3.64 | -13.52 |
| Chronic pulmonary disease | 16.62 | 10.56 | -6.06 | 9.24 | 7.90 | -1.34 | 12.53 | 7.64 | -4.89 | 10.84 | 7.16 | -3.68 | 12.25 | 8.20 | -4.05 |
| Diabetes without complications | 10.79 | 8.92 | -1.87 | 12.15 | 10.59 | -1.56 | 14.09 | 15.03 | 0.94 | 13.69 | 13.54 | -0.15 | 13.92 | 12.76 | -1.16 |
| Solid tumor without metastasis | 7.08 | 2.46 | -4.61 | 4.84 | 2.00 | -2.84 | 5.36 | 2.39 | -2.96 | 9.04 | 4.02 | -5.02 | 4.82 | 1.96 | -2.86 |
| Valvular disease | 6.68 | 2.11 | -4.57 | 2.96 | 0.86 | -2.10 | 4.87 | 1.25 | -3.62 | 5.62 | 2.12 | -3.50 | 2.27 | 1.08 | -1.19 |
| Diabetes with complications | 5.43 | 2.08 | -3.35 | 8.85 | 3.29 | -5.57 | 10.26 | 5.05 | -5.21 | 7.20 | 3.98 | -3.22 | 14.83 | 4.66 | -10.17 |
| Other neurological disorders | 5.38 | 3.31 | -2.07 | 6.09 | 3.23 | -2.85 | 5.48 | 3.23 | -2.25 | 5.18 | 3.20 | -1.98 | 8.28 | 2.98 | -5.30 |
| Metastatic cancer | 5.23 | 1.54 | -3.69 | 3.57 | 1.25 | -2.32 | 3.82 | 1.54 | -2.28 | 7.14 | 2.70 | -4.43 | 3.17 | 1.02 | -2.15 |
| Peripheral vascular disorders | 5.08 | 2.20 | -2.89 | 3.35 | 1.48 | -1.87 | 3.30 | 1.70 | -1.60 | 2.55 | 1.75 | -0.80 | 4.24 | 1.77 | -2.48 |
| Depression | 4.57 | 6.96 | 2.39 | 3.23 | 3.99 | 0.76 | 4.71 | 5.81 | 1.10 | 2.34 | 3.36 | 1.01 | 4.86 | 5.95 | 1.08 |
| Alcohol abuse | 4.49 | 3.02 | -1.47 | 8.98 | 3.10 | -5.88 | 8.14 | 4.26 | -3.88 | 2.79 | 1.60 | -1.19 | 13.50 | 6.66 | -6.83 |
| Hypothyroidism | 4.44 | 5.28 | 0.83 | 1.41 | 1.67 | 0.26 | 2.28 | 3.78 | 1.50 | 2.13 | 3.28 | 1.16 | 2.24 | 3.85 | 1.60 |
| Hypertension (Complications) | 3.93 | 4.84 | 0.91 | 10.13 | 12.82 | 2.69 | 6.61 | 10.03 | 3.43 | 7.74 | 11.85 | 4.11 | 6.52 | 5.78 | -0.74 |
| Renal failure | 3.67 | 7.54 | 3.87 | 8.51 | 11.88 | 3.37 | 6.81 | 11.25 | 4.44 | 10.14 | 14.60 | 4.46 | 7.72 | 9.32 | 1.60 |
| Paralysis | 2.70 | 0.75 | -1.95 | 2.87 | 0.90 | -1.97 | 3.09 | 0.93 | -2.15 | 5.70 | 1.11 | -4.59 | 3.01 | 0.72 | -2.29 |
| Obesity | 2.60 | 4.68 | 2.07 | 3.32 | 5.33 | 2.02 | 2.89 | 5.79 | 2.90 | 1.20 | 3.17 | 1.96 | 3.28 | 5.45 | 2.17 |
| Liver disease | 2.26 | 1.73 | -0.53 | 2.34 | 1.17 | -1.17 | 4.47 | 3.29 | -1.18 | 3.19 | 2.49 | -0.70 | 6.31 | 3.41 | -2.89 |
| Psychoses | 2.11 | 1.06 | -1.05 | 3.00 | 1.94 | -1.06 | 2.96 | 1.62 | -1.34 | 1.42 | 1.22 | -0.20 | 2.08 | 1.01 | -1.08 |
| Coagulopathy | 1.93 | 1.50 | -0.42 | 1.77 | 1.06 | -0.71 | 3.12 | 1.94 | -1.17 | 2.97 | 2.13 | -0.84 | 3.70 | 1.77 | -1.93 |
| Drug abuse | 1.87 | 2.13 | 0.26 | 6.23 | 3.68 | -2.55 | 5.78 | 3.30 | -2.49 | 1.21 | 1.36 | 0.15 | 5.13 | 2.83 | -2.30 |
| Rheumatoid arthritis/collagen vascular diseases | 1.77 | 1.30 | -0.47 | 1.60 | 1.30 | -0.30 | 1.68 | 1.55 | -0.13 | 1.98 | 1.33 | -0.64 | 1.81 | 1.60 | -0.21 |
| Blood loss anemia | 1.43 | 0.35 | -1.09 | 1.35 | 0.40 | -0.94 | 1.54 | 0.48 | -1.06 | 2.27 | 0.50 | -1.76 | 1.16 | 0.38 | -0.79 |
| Deficiency anemia | 1.40 | 0.86 | -0.54 | 2.22 | 1.33 | -0.89 | 1.90 | 1.26 | -0.63 | 1.23 | 1.30 | 0.07 | 1.36 | 1.10 | -0.25 |
| Pulmonary circulation disorders | 1.34 | -0.16 | -1.50 | 0.92 | 0.09 | -0.83 | 0.91 | -0.12 | -1.04 | 1.22 | -0.58 | -1.80 | 1.27 | -0.16 | -1.42 |
| Peptic ulcer disease excluding bleeding | 1.30 | 0.35 | -0.95 | 1.19 | 0.31 | -0.88 | 1.30 | 0.44 | -0.86 | 2.10 | 0.58 | -1.52 | 1.76 | 0.21 | -1.55 |
| Lymphoma | 0.77 | 0.46 | -0.31 | 0.59 | 0.38 | -0.21 | 0.81 | 0.47 | -0.34 | 0.81 | 0.54 | -0.26 | 0.69 | 0.33 | -0.36 |
| AIDS/HIV | 0.54 | 0.09 | -0.45 | 3.37 | 0.71 | -2.66 | 4.12 | 0.40 | -3.72 | 0.49 | 0.10 | -0.39 | 1.75 | 0.11 | -1.64 |

Table S2. Change in rank of comorbidity from 1993 to 2012, by race/ethnicity

| **Comorbidity** | **White** | | | **Black** | | | **Hispanic** | | | **Asian/ Pacific Islander** | | | | **Native American** | | |
| --- | --- | --- | --- | --- | --- | --- | --- | --- | --- | --- | --- | --- | --- | --- | --- | --- |
|  | 1993 | 2012 | ∆ | 1993 | 2012 | ∆ | 1993 | 2012 | ∆ | 1993 | 2012 | ∆ | 1993 | | 2012 | ∆ |
| Hypertension (Uncomplicated) | 1 | 1 | 0 | 1 | 1 | 0 | 1 | 1 | 0 | 2 | 1 | 1 | 2 | | 1 | 1 |
| Fluid and electrolyte disorders | 2 | 2 | 0 | 2 | 4 | -2 | 2 | 3 | -1 | 1 | 2 | -1 | 1 | | 2 | -1 |
| Cardiac arrhythmias | 3 | 4 | -1 | 9 | 7 | 2 | 5 | 7 | -2 | 4 | 6 | -2 | 8 | | 7 | 1 |
| Heart failure | 4 | 11 | -7 | 3 | 9 | -6 | 3 | 16 | -13 | 3 | 8 | -5 | 3 | | 13 | -10 |
| Chronic pulmonary disease | 5 | 3 | 2 | 6 | 6 | 0 | 6 | 6 | 0 | 6 | 7 | -1 | 7 | | 5 | 2 |
| Diabetes without complications | 6 | 5 | 1 | 4 | 5 | -1 | 4 | 2 | 2 | 5 | 4 | 1 | 5 | | 3 | 2 |
| Solid tumor without metastasis | 7 | 14 | -7 | 13 | 15 | -2 | 13 | 17 | -4 | 8 | 9 | -1 | 15 | | 17 | -2 |
| Valvular disease | 8 | 17 | -9 | 20 | 25 | -5 | 14 | 24 | -10 | 13 | 18 | -5 | 21 | | 22 | -1 |
| Diabetes with complications | 9 | 18 | -9 | 8 | 12 | -4 | 7 | 10 | -3 | 10 | 10 | 0 | 4 | | 11 | -7 |
| Other neurological disorders | 10 | 12 | -2 | 12 | 13 | -1 | 12 | 15 | -3 | 14 | 13 | 1 | 9 | | 15 | -6 |
| Metastatic cancer | 11 | 20 | -9 | 14 | 21 | -7 | 18 | 22 | -4 | 11 | 15 | -4 | 19 | | 23 | -4 |
| Peripheral vascular disorders | 12 | 15 | -3 | 16 | 18 | -2 | 19 | 19 | 0 | 18 | 19 | -1 | 16 | | 18 | -2 |
| Depression | 13 | 7 | 6 | 18 | 10 | 8 | 15 | 8 | 7 | 19 | 11 | 8 | 14 | | 8 | 6 |
| Alcohol abuse | 14 | 13 | 1 | 7 | 14 | -7 | 8 | 11 | -3 | 17 | 20 | -3 | 6 | | 6 | 0 |
| Hypothyroidism | 15 | 8 | 7 | 26 | 17 | 9 | 24 | 12 | 12 | 21 | 12 | 9 | 22 | | 12 | 10 |
| Hypertension (Complications) | 16 | 9 | 7 | 5 | 2 | 3 | 10 | 5 | 5 | 9 | 5 | 4 | 11 | | 9 | 2 |
| Renal failure | 17 | 6 | 11 | 10 | 3 | 7 | 9 | 4 | 5 | 7 | 3 | 4 | 10 | | 4 | 6 |
| Paralysis | 18 | 25 | -7 | 21 | 24 | -3 | 21 | 25 | -4 | 12 | 25 | -13 | 20 | | 25 | -5 |
| Obesity | 19 | 10 | 9 | 17 | 8 | 9 | 23 | 9 | 14 | 28 | 14 | 14 | 18 | | 10 | 8 |
| Liver disease | 20 | 19 | 1 | 22 | 22 | 0 | 16 | 14 | 2 | 15 | 16 | -1 | 12 | | 14 | -2 |
| Psychoses | 21 | 23 | -2 | 19 | 16 | 3 | 22 | 20 | 2 | 24 | 24 | 0 | 23 | | 24 | -1 |
| Coagulopathy | 22 | 21 | 1 | 24 | 23 | 1 | 20 | 18 | 2 | 16 | 17 | -1 | 17 | | 19 | -2 |
| Drug abuse | 23 | 16 | 7 | 11 | 11 | 0 | 11 | 13 | -2 | 27 | 21 | 6 | 13 | | 16 | -3 |
| Rheumatoid arthritis/collagen vascular diseases | 24 | 22 | 2 | 25 | 20 | 5 | 26 | 21 | 5 | 23 | 22 | 1 | 24 | | 20 | 4 |
| Blood loss anemia | 25 | 28 | -3 | 27 | 27 | 0 | 27 | 26 | 1 | 20 | 28 | -8 | 29 | | 26 | 3 |
| Deficiency anemia | 26 | 24 | 2 | 23 | 19 | 4 | 25 | 23 | 2 | 25 | 23 | 2 | 27 | | 21 | 6 |
| Pulmonary circulation disorders | 27 | 30 | -3 | 29 | 30 | -1 | 29 | 30 | -1 | 26 | 30 | -4 | 28 | | 30 | -2 |
| Peptic ulcer disease excluding bleeding | 28 | 27 | 1 | 28 | 29 | -1 | 28 | 28 | 0 | 22 | 26 | -4 | 25 | | 28 | -3 |
| Lymphoma | 29 | 26 | 3 | 30 | 28 | 2 | 30 | 27 | 3 | 29 | 27 | 2 | 30 | | 27 | 3 |
| AIDS/HIV | 30 | 29 | 1 | 15 | 26 | -11 | 17 | 29 | -12 | 30 | 29 | 1 | 26 | | 29 | -3 |


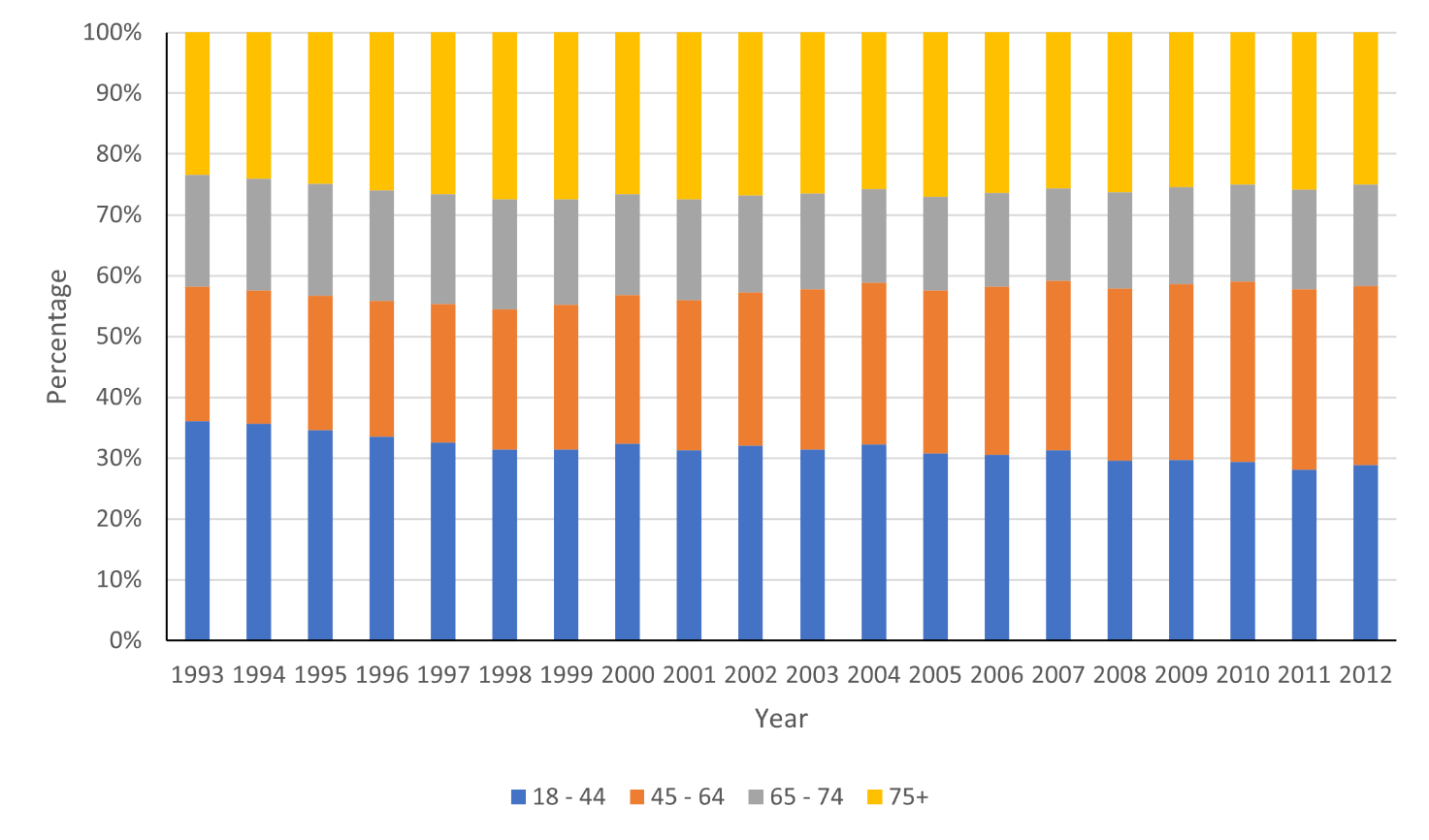


Figure S1. Percentage of hospitalized patients stratified by age category from 1993 to 2012


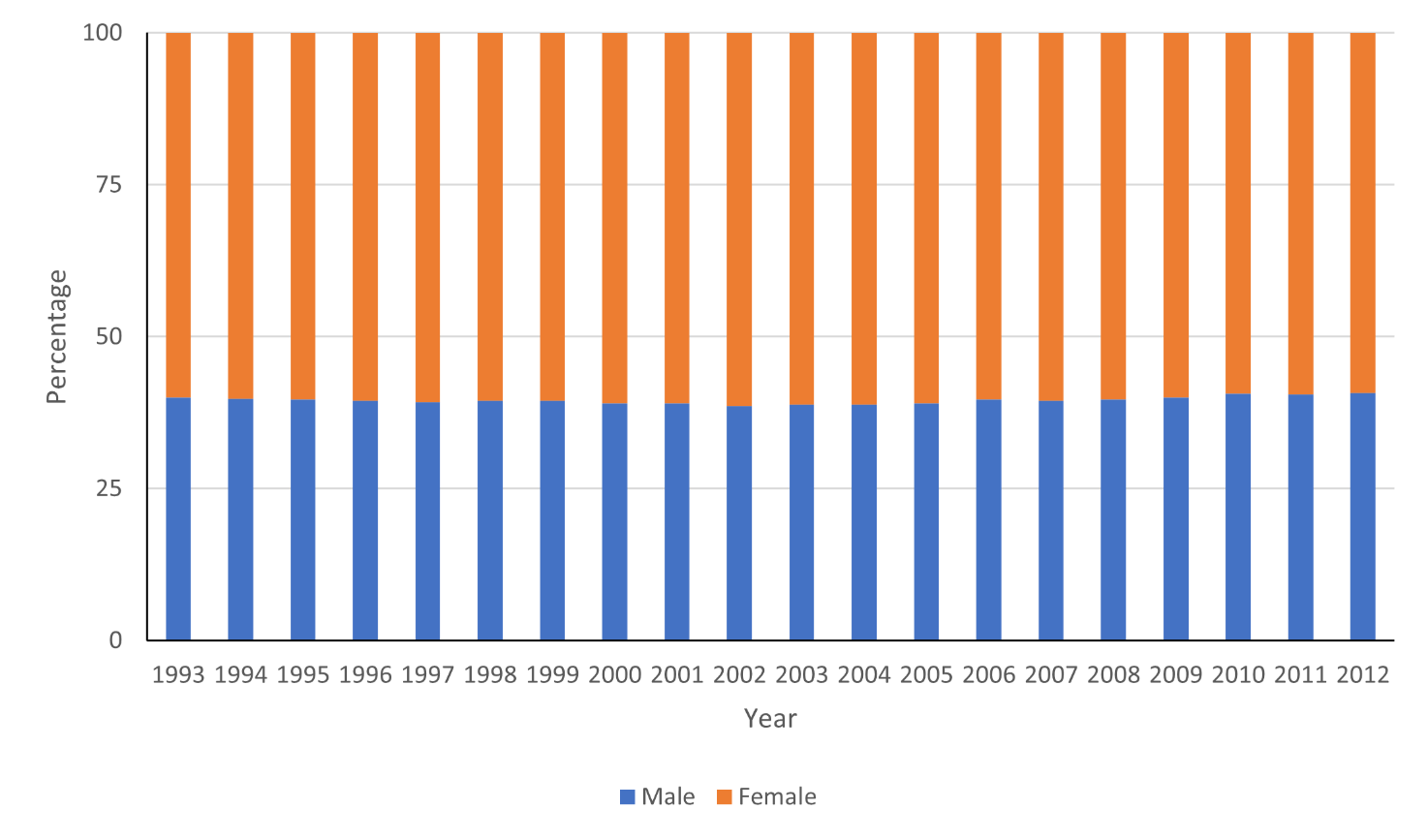


Figure S2. Percentage of hospitalized patients stratified by sex from 1993 to 2012


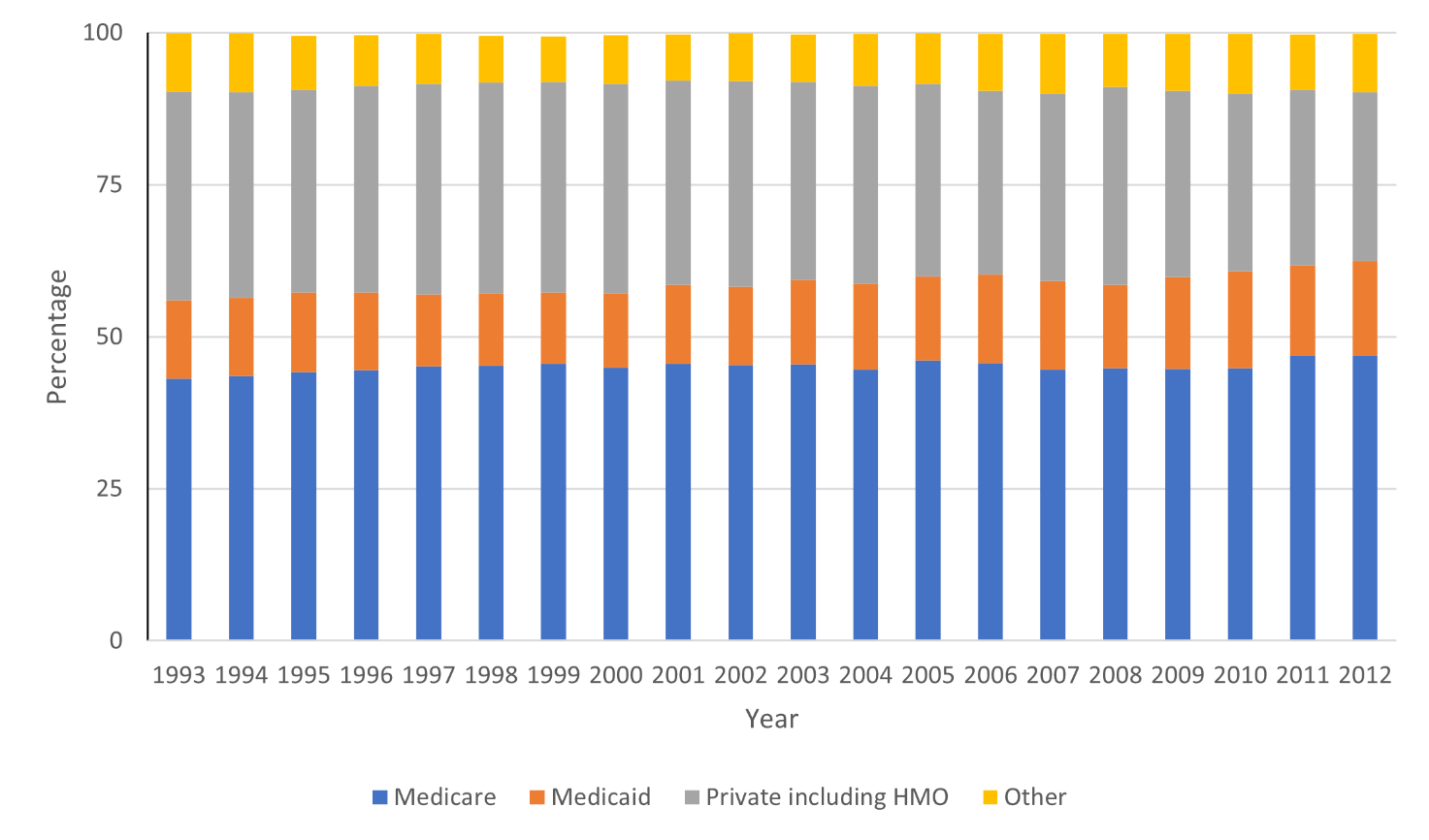


Figure S3. Percentage of hospitalized patients stratified by payer category from 1993 to 2012


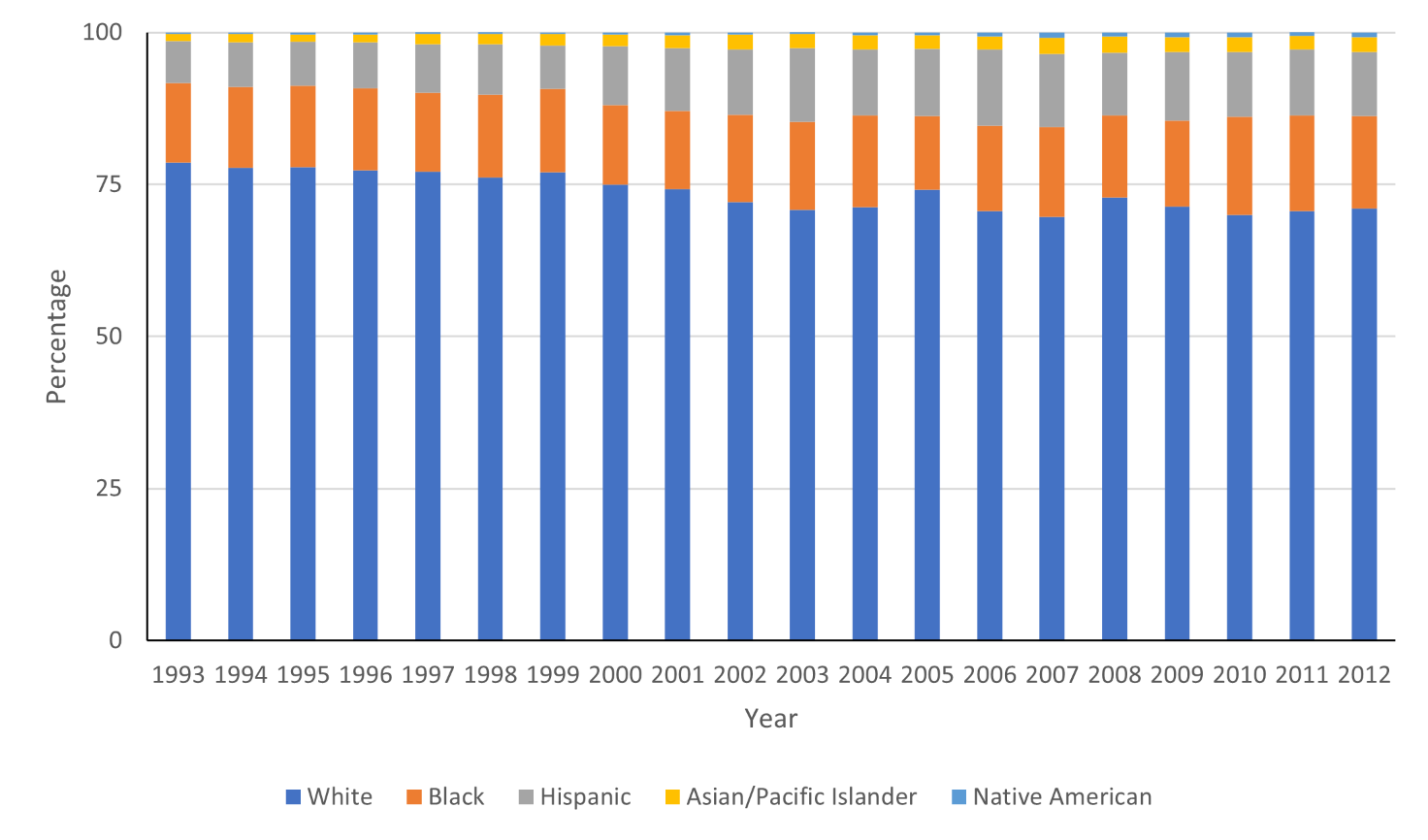


Figure S4. Percentage of hospitalized patients stratified by race/ethnicity from 1993 to 2012


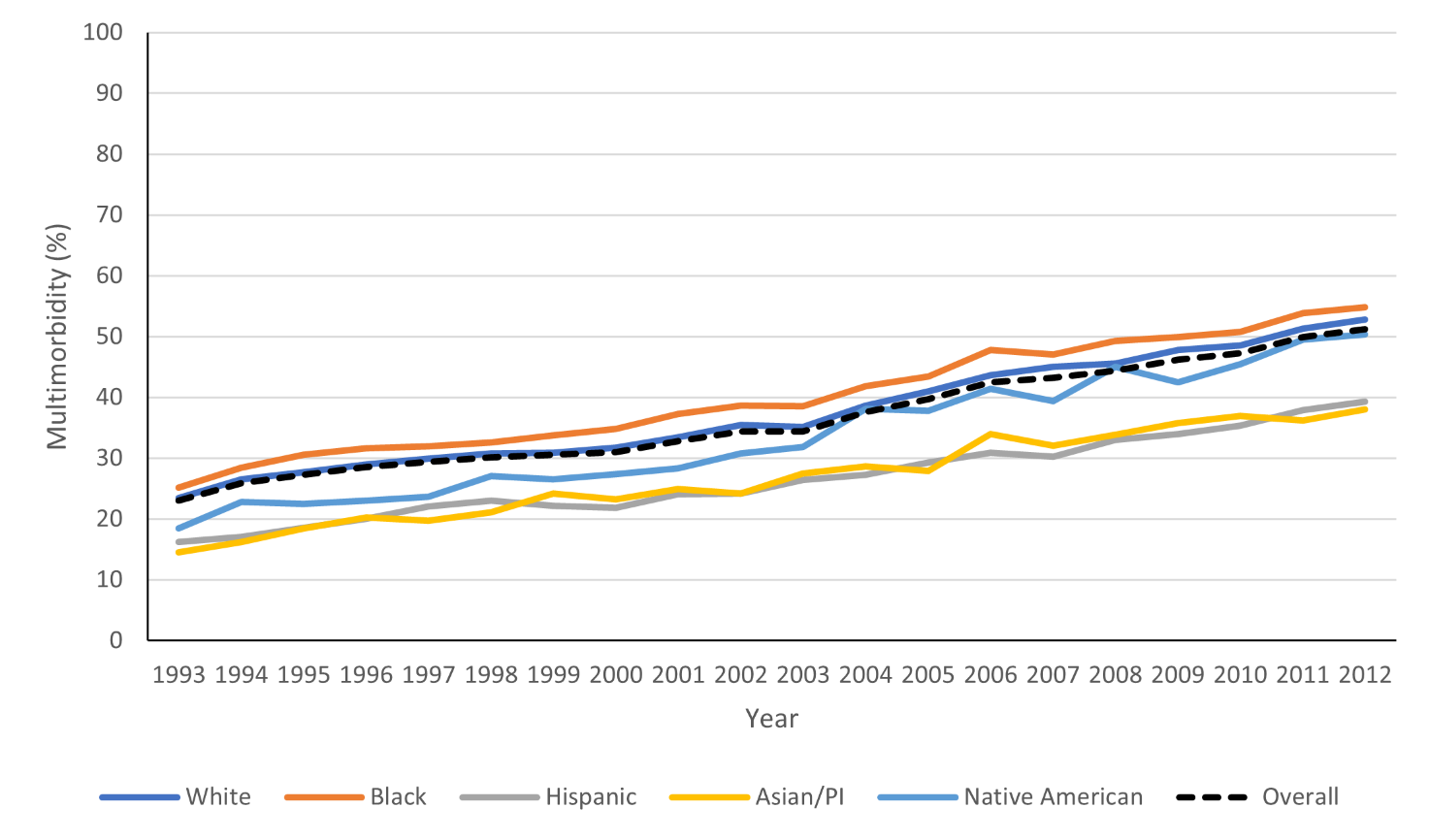


Figure S5. Percentage of hospitalized patients with multimorbiidity in each race/ethnicity category from 1993 to 2012
